# Vegetation cover fraction and the residential distribution of child-rearing households in urban Tokyo and Osaka: An ecological cross-sectional analysis

**DOI:** 10.1371/journal.pone.0345197

**Published:** 2026-03-16

**Authors:** Moe Otani, Daisuke Matsushita

**Affiliations:** Department of Living Environment Design, Graduate School of Human Life and Ecology, Osaka Metropolitan University, Osaka, Japan; The Chinese University of Hong Kong, HONG KONG

## Abstract

In Japan, promoting the long-term settlement of child-rearing households is critical to counteract community decline driven by a falling birthrate and an aging population. Most previous studies on residential location preferences among child-rearing households have relied on self-reported data, and a lack of quantitative evidence based on geospatial and demographic indicators still remains. This ecological cross-sectional study examined the association between vegetation cover fraction (VCF) and the residential distribution of child-rearing households by analyzing the relationship between VCF and the proportion of children (POC) in Tokyo and Osaka. Correlations between VCF and the POC were calculated for all town blocks with VCF values below 0.5 (representing urban and suburban areas rather than forested or rural regions), stratified by distance from the city center and school-age groups, and adjusted for neighborhood environmental and transportation accessibility factors. VCF showed a positive association with the POC even after adjustment (Tokyo: 4,814 town blocks, coefficient: 0.05; Osaka: 7,337 town blocks, coefficient: 0.040). Significant positive associations were observed within 0–15 km from the city center in Tokyo and within 5–15 km in Osaka, but not beyond 15 km. The strongest associations in Tokyo were observed within 5 km for preschool-aged children and 5–10 km for older students, while those in Osaka were noted within 5–10 km for younger children and 20–25 km for junior high school students. These findings suggest that the residential distribution of child-rearing households in urban areas is positively associated with nearby urban greenery.

## Introduction

In Japan, various housing support measures for child-rearing households, such as housing subsidies and tax deductions, have been introduced by municipal governments to mitigate the rapid progression of population decline and aging. Furthermore, many municipalities have launched initiatives to promote the migration of child-rearing households, intensifying competition among cities to attract new residents. Beyond similar levels of economic support offered by municipalities, the development of attractive neighborhood environments and urban design for child-rearing households plays a decisive role in determining success or failure in intercity competition [1]. Previous studies in Japan have reported that child-rearing households prefer areas that offer not only convenient living environments and accessibility for commuting but also abundant urban greenery [2,3]. However, it is important to note that observed residential patterns may reflect not only household preferences but also housing market constraints, school district limitations, zoning regulations, and historical settlement patterns. Furthermore, greener areas may be associated with trade-offs such as higher housing costs, lower accessibility to urban amenities, and longer commuting distances. Our ecological analysis cannot distinguish between these mechanisms but provides valuable descriptive evidence on area-level associations. The positive impacts of urban greenery on residents have attracted widespread global attention. In their systematic review, Syamili et al. (2023) reported that urban greenery contributes to improved happiness and mental health among residents [4]. Urban greenery also influences the physical and psychological well-being of children. Maas et al. (2009) found that urban greenery improves psychological health and happiness, reduces the risk of depression and anxiety, and supports mental well-being of children. [5] Dadvand et al. (2015) reported that access to natural environments promotes healthier growth, cognitive development, and emotional stability among children. [6] Garcia and Lee (2024) suggested that tree loss may negatively impact academic performance. [7] Putra et al. (2021) reported that enhancing the quality of neighborhood green spaces can foster the enjoyment of physical activities, social interaction, mental health, and prosocial behavior of children. [8] Akpinar (2017) reported that proximity to urban greenery is associated with a higher frequency of physical activity and less screen time. [9] Squillacioti et al. (2023) reported that children living in greener, less urbanized environments were less likely to spend more than 15 min sitting daily. [10] Stigsdotter and Grahn (2011) noted that higher stress levels were observed among younger individuals, women, and parents with small children who had poor access to green spaces and that such individuals were more likely to desire more frequent use of public green spaces. [11]Thus, several studies have demonstrated that urban greenery positively influences happiness, psychological health, cognitive development, learning, social behavior, and physical activity among residents, including children and child-rearing households. However, most previous studies on subjective evaluations of urban greenery and its proximity have relied heavily on self-reported data. We identified a research gap regarding the lack of quantitative evidence on the association between urban greenery and residential distribution patterns. It is now possible to quantitatively capture the amount and condition of urban greenery in a region using spectral analysis of multispectral images obtained from remote sensing technologies such as satellites or aircraft, via indices like the normalized difference vegetation index (NDVI) [12]. By analyzing open data on vegetation cover fraction (VCF) along with demographic indicators, neighborhood environmental factors, and transportation accessibility factors, it is possible to examine the spatial patterns of where child-rearing households are concentrated. In this ecological cross-sectional study, we quantitatively investigated the association between urban greenery and the residential distribution of child-rearing households in urban areas based on demographic and geospatial open data. The primary aim was to examine the relationship between VCF and the proportion of children (POC) at the town block level in the urban areas of Tokyo and Osaka, stratified by distance from the city center and school-age groups.

Various factors are considered to be associated with residential location selection. A study by Nishiyama et al. (2011) in Japan reported that residents can be broadly classified into two groups: those who prioritize the neighborhood environment and those who prioritize everyday convenience. [13] We adopted a statistical approach adjusting for neighborhood environmental factors and transportation accessibility factors as assumed covariates to verify whether urban greenery is independently associated with the residential distribution of child-rearing households. Demonstrating that child-rearing households choose areas with abundant urban greenery could provide an empirical basis for urban design strategies aimed at attracting such households.

## Materials and methods

### Study design

We analyzed the correlation between VCF and the POC for all town blocks in the urban areas of Tokyo and Osaka. We then examined whether this correlation remained robust after adjusting for covariates that are presumed to influence residential location preferences, including neighborhood environmental factors (number of reported crimes, proportion of residential zoning districts, and distances to the nearest hospital and school) and transportation accessibility factors (distance from the city center, distance to the nearest station, and station ridership). The proportion of children (POC) was defined as the ratio of the combined population of preschool-aged children (ages 0–5), elementary school students (ages 6–11), and junior high school students (ages 12–14) to the total population in each town block. This measure captures the relative child density while accounting for overall population size.

The study addressed the following three objectives sequentially.

For Objective 1, we categorized the continuous variables of VCF, neighborhood environmental factors, and transportation accessibility factors into tercile-based categories for each town block in Tokyo and Osaka. The tercile approach was used to provide an intuitive, interpretable visualization of dose-response patterns across low, medium, and high categories, facilitating comparison of effect magnitudes across different predictors. We then independently assessed the contribution of each factor to the POC by analyzing their correlations.

For Objective 2, we analyzed the correlation between VCF and the POC for each town block in Tokyo and Osaka and assessed whether the correlation remained significant after adjusting for the assumed covariates. These multivariable linear regression models with continuous POC serve as the primary inferential analyses, as they preserve the continuous nature of the outcome variable and avoid information loss from dichotomization.

For Objective 3, we similarly calculated the correlation between VCF and the POC after adjusting for the assumed covariates and conducted stratified analyses to examine how the correlation varied by distance from the city center (<5 km, 5–10 km, 10–15 km, 15–20 km, 20–25 km, > 25 km) and by school-age groups. The distance thresholds were selected based on the urban structure of Japanese metropolitan areas: the 5-km zone corresponds to the urban core (inside loop railway lines such as the Yamanote Line in Tokyo and the Osaka Loop Line); the 5–10 km zone represents the inner suburban ring with mixed residential and commercial development; and the 10–15 km zone encompasses the outer suburban area with predominantly residential land use.

In addition to stratified analyses, we formally tested interaction effects by including VCF × Distance zone and VCF × Age group interaction terms in the main regression models to assess whether the VCF-POC relationship varied significantly across these subgroups.

### Data collection

We collected the latest open datasets on demographic and geospatial information, as summarized in Table 1, and prepared them for analysis through necessary corrections and processing. The details of each dataset are described in the following sections.

**Table 1:** Datasets.

Datasets	Data sources
**Demographic data**	
Population of preschoolers, elementary school students, and junior high school students	e-Stat Census – 2020 Census – Subregional Aggregation – 13. Tokyo, 27. Osaka Prefecture – Population by Type of Male and Female, Enrolled and Unenrolled – Chome, Aza, etc. [14,15] https://www.e-stat.go.jp/stat-search/files?tclass=000001147875&cycle=0 https://www.e-stat.go.jp/stat-search/files?tclass=000001147861&cycle=0
**Geospatial data**	
Geographic coordinates, area, boundary	e-Stat, Boundary Data Download −2020 – Subregion (Chome, Aza, etc.) JGD2000 – World Geodetic System Plane-Right Coordinate System Shapefile – All areas of 13 Tokyo, 27 Osaka [16,17] https://www.e-stat.go.jp/gis/statmap-search/data?dlserveyId=A002005212020&code=13&coordSys=1&format=shape&downloadType=5&datum=2000
Vegetation cover fraction	Kiyono Tomoki, Fujiwara Kunihiko, & Tsurumi Ryuta. (2021). Vegetation cover fraction in each town block across Japan (1.0.1) [Data set] [18] https://doi.org/10.5281/zenodo.5553516
Number of crimes in Osaka	Osaka Prefectural Police, Open Data Site, Crime Incidents in 2020 [19] https://www.police.pref.osaka.lg.jp/seikatsu/9290.html
Number of crimes in Tokyo	Tokyo Metropolitan Police Department, Number of reported cases by town, type of crime, and modus operandi in 2020 [20] https://www.keishicho.metro.tokyo.lg.jp/about_mpd/jokyo_tokei/jokyo/ninchikensu.html
Zoning district boundary	The Ministry of Land, Infrastructure, Transport and Tourism, National Land Numerical Information Application Area Data 2011, Tokyo, Osaka [21] https://nlftp.mlit.go.jp/ksj/gml/datalist/KsjTmplt-A29.html
Station latitude and longitude, number of passengers getting on and off	The Ministry of Land, Infrastructure, Transport and Tourism, National Land Numerical Information, Number of passengers boarding and alighting by station, Japan, 2021 [22] https://nlftp.mlit.go.jp/ksj/gml/datalist/KsjTmplt-S12-2022.html
School latitude and longitude	The Ministry of Land, Infrastructure, Transport and Tourism, National Land Numerical Information School Data, Tokyo, Osaka, 2021 [23] https://nlftp.mlit.go.jp/ksj/gml/datalist/KsjTmplt-P29-2023.html
Hospital latitude and longitude	The Ministry of Land, Infrastructure, Transport and Tourism, National Land Information Medical Institution Data, Tokyo, Osaka, 2014 [24] https://nlftp.mlit.go.jp/ksj/gml/datalist/KsjTmplt-P04-v2_1.html

### VCF

The NDVI is a widely used indicator to assess vegetation quantity and health status and is calculated based on the reflectance of near-infrared (NIR) and red light, defined by the following formula:


$$NDVI\;=\;\frac{\left(NIR\;-\;Red\right)}{\left(NIR\;+\;Red\right)}$$ (1)

where NIR and Red refer to reflectance in the near-infrared spectrum and in the red spectrum, respectively.

NDVI values range from −1–1 and are interpreted in relation to vegetation as follows:

Values below 0.2 indicate areas with little to no vegetation, such as soil or urban surfaces.

Values between 0.2 and 0.5 indicate areas with grasslands or low-density vegetation.

Values above 0.5 indicate areas with dense and healthy vegetation, such as forests [25].

**Fig 1:**
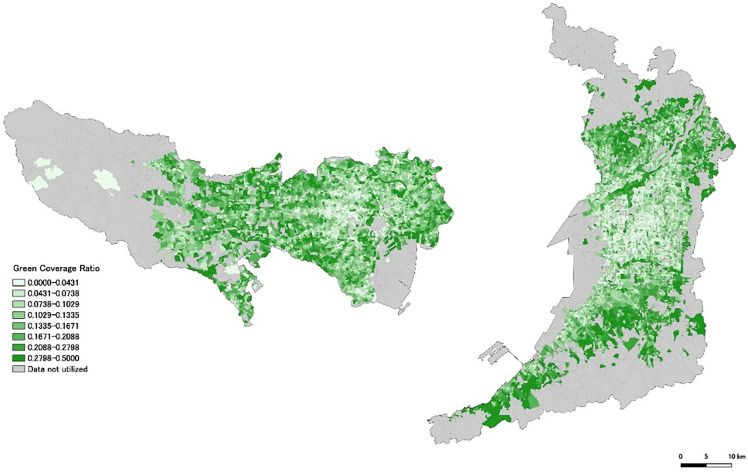
Distribution of VCF. Source: National Land Numerical Information (Administrative Boundaries Data), Ministry of Land, Infrastructure, Transport and Tourism of Japan (MLIT), which is free to use under Article 13 of the Japanese Copyright Act (public domain).

In this study, we used a VCF dataset created and published by Kiyono et al. [18]. This VCF dataset was developed by calibrating Sentinel-2 satellite imagery over the Tokyo metropolitan area using Google Earth Engine. This dataset offers higher accuracy than NDVI alone, with an average absolute error of approximately 2% in urban areas of Japan. This study focuses on urban areas; therefore, records with a VCF greater than 0.5 were excluded from both Tokyo and Osaka datasets. According to NDVI interpretation standards and the original VCF dataset documentation, VCF values above 0.5 typically indicate dense vegetation characteristic of forests, rural areas, or conservation green spaces rather than urban residential environments. Fig 1 shows the distribution of VCF for each town block in Tokyo and Osaka, expressed in eight gradations. The figure was generated using the open-source geographic information system QGIS (QGIS.org, 2025, QGIS Geographic Information System, QGIS Association) and town block boundary data from e-Stat. [16,17].

### Demographic–geospatial–VCF dataset

#### Creation of the demographic–geospatial dataset.

**Fig 2:**
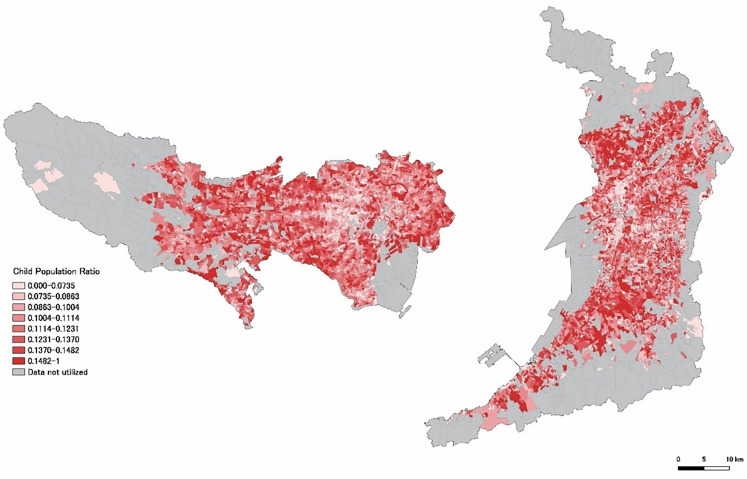
Distribution of the POC. Source: National Land Numerical Information (Administrative Boundaries Data), Ministry of Land, Infrastructure, Transport and Tourism of Japan (MLIT), which is free to use under Article 13 of the Japanese Copyright Act (public domain).

**Fig 3:**
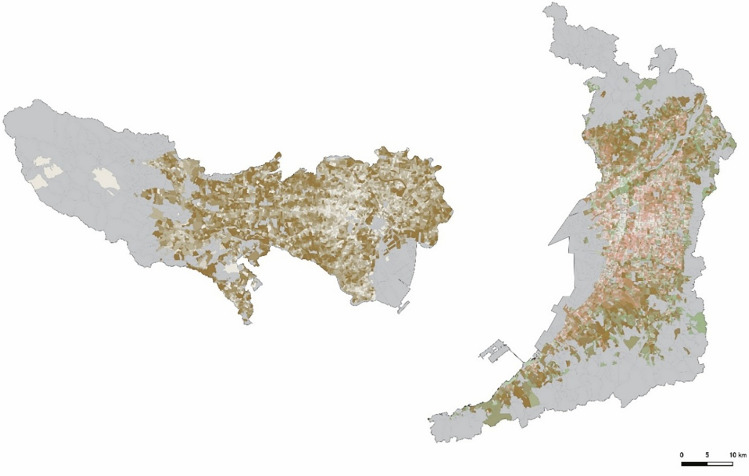
Superposition of VCF and the POC. Source: National Land Numerical Information (Administrative Boundaries Data), Ministry of Land, Infrastructure, Transport and Tourism of Japan (MLIT), which is free to use under Article 13 of the Japanese Copyright Act (public domain).

From the 2020 Population Census data (20,604 records for Tokyo and 31,851 records for Osaka; e-Stat, 2020a, 2020b), we excluded gender-specific data and data corresponding to aggregated or masked regions. A new field called “KEY_CODE” was created by combining the municipality code and the town block code, serving as a link between the demographic and the GIS boundary data. Using the KEY_CODE field, we linked the demographic data with geospatial data to create a demographic–geospatial dataset. Fig 2 shows the distribution of the POC, expressed in eight gradations similar to the VCF shown in Fig 1. Fig 3 was generated by overlaying the VCF (Fig 1) and the POC (Fig 2) based on subtractive color mixing.

#### Correction of the VCF dataset.

Since the town block codes in the VCF dataset (5,519 records for Tokyo and 8,689 records for Osaka; [18] were not fully consistent with the KEY_CODE format, corrections were made as follows. Arabic numerals in the “S_NAME” field (town block name) of the VCF dataset were converted into Kanji numerals to match the demographic–geospatial dataset. The “CITY_NAME” (municipality name) and “S_NAME” were then combined to create a new field, i.e., “municipality and town block name.” In the designated cities of Osaka and Sakai, ward names were recorded instead of city names in the CITY_NAME field, so the city names were supplemented. All town block names were checked manually to identify mismatches between the S_AREA (town block code) in the VCF dataset and the town block codes in the demographic–geospatial dataset. The corresponding town block code was appended to ensure uniqueness in cases where town block names were duplicated.

#### Correction of the demographic–geospatial dataset.

Records with missing total population values were removed from the demographic–geospatial dataset. Following the same procedure as that for the VCF dataset, a “municipality and town block name” field was created by merging the fields of municipality name and the large-area town name. In cases of duplication of names, the corresponding town block code was appended to create unique identifiers.

#### Creation of the demographic–geospatial–VCF dataset.

Finally, the demographic–geospatial data and the VCF data were linked based on the “municipality and town block name” field, completing the preparation of the demographic–geospatial–VCF dataset.

However, island regions, such as the Ogasawara Islands, which are part of Tokyo but have population densities and social characteristics that differ significantly from urban areas, were excluded from the analysis. In Japan, the relationship between VCF and land use is generally as follows:

VCF < 0.2: Urban core areas
VCF 0.2–0.5: Suburban areas
VCF 0.5–0.7: Rural or hilly areas and low-development zones
VCF > 0.7: Forested regions, mountainous areas, and conservation green spaces

This study focuses on urban areas; therefore, records with a VCF greater than 0.5 were excluded from both Tokyo and Osaka datasets.

We calculated the distance from each town block to the city center to classify town blocks by distance from the city center. JR Tokyo Station (latitude 35.681236°, longitude 139.767125°) and JR Osaka Station (latitude 34.7024°, longitude 135.4959°) were set as the city centers for Tokyo and Osaka, respectively. The calculated distances were added to the dataset as the “distance from the city center.” We approximated the Earth as a sphere with a radius of 6,370 km and calculated the great-circle distance between two points based on their latitudes and longitudes.

Given two points (φ1, λ1) and (φ2, λ2), L [km] is calculated as follows [26]:


L = 6370 arccos(sin φ1 sin φ2 + cos φ1 cos φ2 cos(λ1 − λ2)) (2)

### Crime incidents

The number of crime incidents is an important factor that represents neighborhood environmental safety [27]. For Tokyo, we used data from the Tokyo Metropolitan Police Department on Number of reported cases by town, type of crime, and modus operandi in 2020 [20]. For Osaka, we used data from the Osaka Prefectural Police on theft incidents (including vehicle theft, motorcycle theft, bicycle theft, theft from vehicles, theft from vending machines, and purse snatching) by town block [19]. It should be noted that crime data definitions differ between Tokyo (all reported crimes) and Osaka (theft-related incidents only), which may affect cross-city comparisons. The number of crimes for each town block was adjusted according to the population size. These crime datasets did not contain town block codes; therefore, we linked them to the demographic–geospatial–VCF dataset based on the town block name fields. Arabic numerals in the crime data town block names were converted to Kanji numerals to ensure consistency, and names were checked and corrected as necessary. Following these procedures, the crime incident dataset was prepared.

### Residential zoning districts

Residential zoning districts (including first-class low-rise residential zones, second-class low-rise residential zones, first-class mid/high-rise residential zones, second-class mid/high-rise residential zones, first-class residential zones, second-class residential zones, and semiresidential zones) are designated under the City Planning Act to secure and maintain favorable residential environments and thus represent an important factor indicating neighborhood quality. We created a GIS layer by integrating the boundaries of residential zoning districts [21] and calculated the area of residential zoning districts within each town block boundary [16,17]. The proportion of residential zoning area for each town block was calculated using Eq. (3):


$$R_{i}\;=\;\frac{{Sr}_{i}}{{Sc}_{i}}$$ (3)

where $R_{i}$ represents the proportion of residential zoning area in town block, ${Sr}_{i}$ denotes the area of residential zoning districts within town block, and ${Sc}_{i}$ is the total area of town block.

The residential zoning dataset was linked to the demographic–geospatial–VCF dataset based on the common town block code (KEY_CODE) field. Following these procedures, the residential zoning proportion dataset was prepared.

### Stations, schools, and hospitals

Key public facilities, such as stations, schools, and hospitals, are important factors that reflect neighborhood environments and transportation accessibility. For stations, we used the 2021 National Land Numerical Information on station ridership, excluding stations with zero daily ridership [22]. For schools, we used the elementary and junior high school data from the 2021 National Land Numerical Information school dataset [23].

For hospitals, we used the “hospital” category data from the 2021 National Land Numerical Information on medical facilities (The Ministry of Land, Infrastructure, Transport and Tourism, 2014). The distances from the centroid of each town block to the nearest station, school, and hospital were calculated. These datasets were linked to the demographic–geospatial–VCF dataset based on the common town block code (KEY_CODE) field. Following these procedures, the nearest station, school, and hospital datasets were prepared.

### Statistical analysis

We conducted the following statistical analyses corresponding to three objectives.

For Objective 1, we created independent bivariate logistic regression models for each town block in Tokyo and Osaka to predict whether the POC exceeded the median value. This exploratory analysis was used to provide an intuitive visualization of how each predictor differentiates between high and low POC blocks, though we acknowledge that dichotomization results in some information loss. Specifically, the dependent variable was the binarized POC (1, higher than the median; 0, lower than the median), and the independent variables were the tercile-categorized VCF and covariates. Distance from the city center was categorized into six groups (<5 km, 5–10 km, 10–15 km, 15–20 km, 20–25 km, and >25 km) based on the distance from Tokyo Station or Osaka Station. Other covariates were categorized into terciles based on the 33.3rd and 66.7th percentiles. Unadjusted odds ratios for each category were examined.

For Objective 2, we calculated the correlation between the POC and VCF for each town block in Tokyo and Osaka and examined this correlation using three sequential multivariable linear regression models adjusting for neighborhood environmental factors and transportation accessibility factors. Model fit was assessed using R², adjusted R², and root mean square error (RMSE). Variance inflation factors (VIF) were calculated to assess multicollinearity. To assess potential spatial autocorrelation in the regression residuals, we calculated Moran’s I statistics using queen contiguity weights based on town block boundaries.

For Objective 3, we similarly constructed multivariable linear regression models adjusting for the assumed covariates to examine the correlation between the POC and VCF for each town block in Tokyo and Osaka. We then conducted stratified analyses to investigate how the correlation varied by distance from the city center (<5 km, 5–10 km, 10–15 km, 15–20 km, 20–25 km, and >25 km) and by school-age groups (preschool-aged children, elementary school students, and junior high school students). All statistical analyses were conducted using JMP Pro, Version 18.0.2 (SAS Institute Inc., Cary, NC, 1989–2025).

## Results

### Objective 1: Independent bivariate logistic regression models for the POC and VCF, neighborhood environmental factors, and transportation accessibility factors

A total of 4,814 and 7,337 town blocks in Tokyo and Osaka, respectively, were included in the analysis. Table 2 presents the unadjusted odds ratios for each variable category in predicting whether the POC exceeded the median.

**Table 2:** Independent bivariate logistic regression models evaluating the POC (>0.1113), VCF, distance from the city center, neighborhood environment, and transportation accessibility.

		Tokyo (n = 4814)				Osaka (n = 7337)			
		n (%)	Mean (SD)	Unadjusted OR (95% CI)	p	n (%)	Mean (SD)	Unadjusted OR (95% CI)	p
**Proportion of children (POC)**			0.11 (0.035)				0.12 (0.048)		
Preschool-aged children			0.049 (0.017)				0.047 (0.025)		
Elementary school students			0.044 (0.016)				0.046 (0.023)		
Junior high school students			0.022 (0.0088)				0.025 (0.013)		
**Vegetation cover fraction (VCF)**			0.18 (0.097)				0.13 (0.10)		
Low	<0.09297	815 (17%)	0.061 (0.023)	1 (reference)		3195 (44%)	0.049 (0.025)	1 (reference)	
Medium	0.0927–0.1804	1904 (40%)	0.14 (0.025)	2.25 (1.88–2.69)	<0.0001	2227 (30%)	0.13 (0.025)	1.60 (1.44–1.79)	<0.0001
High	0.1804–0.5000	2095 (44%)	0.27 (0.076)	4.40 (3.69–5.26)	<0.0001	1915 (26%)	0.28 (0.080)	1.58 (1.41–1.77)	<0.0001
**Distance zones from the city center**									
0–5 km		608 (13%)	3.24 (1.09)	1 (reference)		731 (10%)	3.24 (1.20)	1 (reference)	
5–10 km		944 (20%)	7.77 (1.46)	0.78 (0.63–0.97)	0.024	1481 (20%)	7.61 (1.45)	1.90 (1.58–2.29)	<0.0001
10–15 km		1198 (25%)	12.3 (1.39)	1.55 (1.27–1.89)	<0.0001	2158 (29%)	12.7 (1.42)	2.14 (1.79–2.55)	<0.0001
15–20 km		439 (9%)	17.3 (1.52)	2.44 (1.90–3.14)	<0.0001	1363 (19%)	17.2 (1.46)	2.79 (2.31–3.36)	<0.0001
20–25 km		349 (7%)	22.5 (1.53)	3.11 (2.36–4.09)	<0.0001	848 (12%)	22.6 (1.46)	2.38 (1.94–2.93)	<0.0001
>25 km		1276 (27%)	33.9 (5.94)	2.29 (1.88–2.79)	<0.0001	756 (10%)	32.9 (7.14)	2.28 (1.84–2.81)	<0.0001
**Crime rate**									
Low	<0.00145	560 (12%)	0.00062 (0.00056)	1 (reference)		3465 (47%)	0.00033 (0.00048)	1 (reference)	
Medium	0.00145–0.00407	1973 (41%)	0.0028 (0.00072)	0.84 (0.69–1.02)	0.077	2151 (29%)	0.0026 (0.00073)	0.82 (0.74–0.92)	0.0004
High	>0.00407	2281 (47%)	0.017 (0.081)	0.34 (0.28–0.41)	<0.0001	1721 (23%)	0.014 (0.051)	0.39 (0.35–0.44)	<0.0001
**Proportion of residential zoning**									
Low	<0.5517	1497 (31%)	0.16 (0.20)	1 (reference)		2513 (34%)	0.14 (0.19)	1 (reference)	
Medium	0.5517–0.9566	1967 (41%)	0.80 (0.11)	1.43 (1.25–1.64)	<0.0001	2165 (30%)	0.79 (0.11)	1.29 (1.15–1.45)	<0.0001
High	>0.9566	1350 (28%)	0.99 (0.012)	4.28 (3.66–5.02)	<0.0001	2659 (36%)	0.99 (0.010)	1.99 (1.79–2.23)	<0.0001
**Distance to the nearest station (m)**									
Low	<391.79 m	2770 (58%)	240.6 (92.4)	1 (reference)		2240 (31%)	236.6 (90.7)	1 (reference)	
Medium	391.79–803.07 m	1597 (33%)	581.2 (117.5)	2.55 (2.21–2.93)	<0.0001	2534 (35%)	583.3 (116.8)	1.93 (1.71–2.16)	<0.0001
High	>803.066 m	1447 (30%)	1271.9 (477.1)	6.08 (5.21–7.08)	<0.0001	2563 (35%)	1403.8 (651.1)	2.19 (1.95–2.46)	<0.0001
**Distance to the nearest school(m)**									
Low	<180.86 m	1801 (37%)	156.8 (63.3)	1 (reference)		2210 (30%)	159.5 (63.6)	1 (reference)	
Medium	180.86–316.78 m	1672 (35%)	331.9 (45.8)	1.01 (0.88–1.15)	0.90	2459 (34%)	331.8 (45.4)	0.92 (0.82–1.04)	0.17
High	>316.78 m	1341 (28%)	569.2 (231.6)	1.17 (1.02–1.35)	<0.0001	2668 (36%)	610.6 (235.6)	0.82 (0.73–0.92)	0.0007
**Distance to the nearest hospital (m)**									
Low	<466.12 m	1581 (33%)	292.5 (108.8)	1 (reference)		2429 (33%)	295.0 (112.2)	1 (reference)	
Medium	466.12–822.05 m	1639 (34%)	635.7 (101.7)	1.42 (1.24–1.64)	<0.0001	2492 (34%)	635.9 (102.2)	1.34 (1.19–1.49)	<0.0001
High	>822.05 m	1594 (33%)	1209.9 (386.3)	2.41 (2.09–2.78)	<0.0001	2416 (33%)	1284.3 (686.1)	1.30 (1.16–1.45)	<0.0001
**Station ridership (passengers per day)**									
Low	<11833	896 (19%)	7253 (2984)	1 (reference)		3112 (42%)	5940 (3448)	1 (reference)	
Medium	11833–28038	1473 (31%)	19,902 (4542)	0.87 (0.73–1.02)	0.093	2614 (36%)	17,704 (4091)	1.06 (0.95–1.17)	0.31
High	>28038	2445 (51%)	69,106 (58945)	0.81 (0.69–0.94)	0.0061	1611 (22%)	50,307 (38813)	1.02 (0.90–1.15)	0.77

Notes: Proportion of children: Ratio of the population of preschool-aged children, elementary school students, and junior high school students to the total population.

Vegetation cover fraction (VCF): Proportion of a town block surface that is covered by live vegetation.

Distance from the city center: Distance from the city center (Tokyo Station or Osaka Station).

Number of crime incidents: Number of reported crimes per capita.

Proportion of residential zoning: Proportion of a town block area designated as residential zoning.

Distance to the nearest station, school, or hospital: Distance from the centroid of a town block to the nearest facility.

Station ridership: Average daily number of passengers at the nearest station.

Significant correlations were observed between the POC and VCF, distance from the city center, number of crime incidents, proportion of residential zoning, and distances to the nearest station, school, and hospital in both Tokyo and Osaka.

For VCF, in Tokyo, the unadjusted odds ratios were significantly higher for medium (the odds ratio [OR]=2.25) and high (OR = 4.40) VCF categories compared with that for low VCF, showing a positive association between higher VCF and higher odds. In Osaka, the odds ratios were also significantly higher for medium (OR = 1.60) and high (OR = 1.58) VCF categories compared with that for low VCF, although the difference between the medium and high categories was smaller than that observed in Tokyo.

Regarding distance from the city center, the highest odds ratio was observed at 20–25 km in Tokyo (OR = 3.11) and at 15–20 km in Osaka (OR = 2.79). Both cities exhibited a unimodal distribution with a peak in odds ratios at these distance categories.

For the number of crime incidents, both Tokyo (OR = 0.34) and Osaka (OR = 0.39) showed a significant negative association, indicating that town blocks with higher crime rates had significantly lower odds ratios.

For the proportion of residential zoning, a positive association was observed in both Tokyo and Osaka, with higher proportions linked to significantly higher odds ratios. In particular, town blocks with a proportion of residential zoning above 0.96 had substantially higher odds ratios in Tokyo (OR = 4.28) compared with those in Osaka (OR = 1.99).

Distance to the nearest station showed a positive association in both Tokyo and Osaka, with longer distances associated with significantly higher odds ratios. In particular, for town blocks located more than 800 m from the nearest station, the odds ratio in Tokyo (OR = 6.08) was much higher than that in Osaka (OR = 2.19).

For distance to the nearest school, longer distances were associated with a higher odds ratio in Tokyo (OR = 1.17), whereas a longer distance was associated with a lower odds ratio in Osaka (OR = 0.82).

For distance to the nearest hospital, Tokyo showed a significant positive association, with longer distances linked to higher odds ratios. In Osaka, the odds ratios were significantly higher in the medium (OR = 1.34)- and long (OR = 1.30)-distance categories compared with that in the short-distance category, although the difference between medium and long categories was smaller than that in Tokyo.

Finally, for station ridership, a higher ridership category was associated with a significantly lower odds ratio in Tokyo (OR = 0.81), while no statistically significant association was observed in Osaka.

### Objective 2: Multivariable linear regression models for the POC and VCF adjusted for neighborhood environmental and transportation accessibility factors

As presented in Table 3, we constructed multivariable linear regression models with the POC in each town block as the dependent variable. Variance inflation factors for all models were below 1.6, indicating that multicollinearity was not a concern. In both Tokyo and Osaka, a significant positive association between the POC and VCF remained even after adjusting for the assumed covariates (Model 3: Tokyo, coefficient: 0.05, 95% CI: 0.039–0.061; Osaka, coefficient: 0.04, 95% CI: 0.028–0.052). This implies that a one-point increase in VCF corresponds to a 0.05-point increase in the POC in Tokyo and a 0.04-point increase in Osaka.

**Table 3:** Nested multivariable linear regression models predicting POC.

	Proportion of children (POC)					
	Model 1		Model 2		Model 3	
	*Coefficient*(95% CI)	*p*	*Coefficient*(95% CI)	*p*	*Coefficient*(95% CI)	*p*
**Tokyo**						
VCF	0.083 (0.072–0.094)	<0.0001	8.2 × 10 ^−^ ⁵ (−1.6 × 10 ^−^ ⁵–2.0 × 10 ^−^ ⁴)	<0.0001	0.050 (0.039–0.061)	<0.0001
Distance to center	0.0002 (0.0001–0.0003)	<0.0001	−0.055 (−0.072– − 0.038)	0.10	−0.0001 (−0.0002– − 3.4 × 10 ^−^ ⁵)	0.0083
Crime rate			0.012 (0.0093–0.015)	<0.0001	−0.044 (−0.061– − 0.028)	<0.0001
Proportion of residential zoning			8.7 × 10 ^−^ ⁶ (6.4 × 10 ^−^ ⁶–1.1 × 10 ^−^ ⁵)	<0.0001	0.0091 (0.0062–0.012)	<0.0001
Distance to the nearest hospital			−1.3 × 10 ^−^ ⁵ (−1.7 × 10 ^−^ ⁵– − 7.8 × 10 ^−^ ⁶)	<0.0001	6.3 × 10 ^−^ ⁶ (4.1 × 10 ^−^ ⁶–8.6 × 10 ^−^ ⁶)	<0.0001
Distance to the nearest school			8.2 × 10 ^−^ ⁵ (−1.6 × 10 ^−^ ⁵–2.0 × 10 ^−^ ⁴)	<0.0001	−1.6 × 10 ^−^ ⁵ (−2.1 × 10 ^−^ ⁵– − 1.2 × 10 ^−^ ⁵)	<0.0001
Distance to the nearest station					1.7 × 10 ^−^ ⁵ (1.5 × 10 ^−^ ⁵–1.9 × 10 ^−^ ⁵)	<0.0001
Station ridership					−4.0 × 10 ^−^ ⁸ (−5.9 × 10 ^−^ ⁸– − 2.1 × 10 ^−^ ⁸)	<0.0001
**Osaka**						
VCF	0.048 (0.037–0.060)	<0.0001	0.055 (0.044–0.067)	<0.0001	0.040 (0.028–0.052)	<0.0001
Distance to center	0.0005 (0.0003–0.0006)	<0.0001	0.0005 (0.0003–0.0006)	<0.0001	0.0004 (0.0002–0.0005)	<0.0001
Crime rate			−0.088 (−0.13– − 0.045)	<0.0001	−0.070 (−0.11– − 0.026)	0.0017
Proportion of residential zoning			0.014 (0.0112–0.017)	<0.0001	0.014 (0.011–0.016)	<0.0001
Distance to the nearest hospital			−2.3 × 10 ^−^ ⁶ (−4.3 × 10 ^−^ ⁶– − 1.7 × 10 ^−^ ⁷)	0.034	−6.3 × 10 ^−^ ⁶ (−8.5 × 10 ^−^ ⁶– − 4.1 × 10 ^−^ ⁶)	<0.0001
Distance to the nearest school			−1.4 × 10 ^−^ ⁵ (−1.9 × 10 ^−^ ⁵– − 9.3 × 10 ^−^ ⁶)	<0.0001	−1.7 × 10 ^−^ ⁵ (−2.2 × 10 ^−^ ⁵– − 1.2 × 10 ^−^ ⁵)	<0.0001
Distance to the nearest station					1.2 × 10 ^−^ ⁵ (9.6 × 10 ^−^ ⁶–1.4 × 10 ^−^ ⁵)	<0.0001
Station ridership					−3.4 × 10 ^−^ ⁸ (−7.9 × 10 ⁻ ⁸–1.1 × 10 ⁻ ⁸)	0.14

For distance from the city center, a negative association was observed in Tokyo (coefficient: −0.0001), while a positive association was observed in Osaka (coefficient: 0.0004). However, as shown in the previous section, the highest odds ratios for having a POC above the median were observed in the 20–25-km zone and the 15–20-km zone for Tokyo and Osaka, respectively, suggesting a unimodal distribution. Therefore, in the next section, we conduct stratified analyses based on distance from the city center.

For the number of crime incidents, significant negative associations with the POC were observed in both Tokyo (coefficient: −0.044) and Osaka (coefficient: −0.070).

For the proportion of residential zoning area, significant positive associations with the POC were observed in both Tokyo (coefficient: 0.0091) and Osaka (coefficient: 0.014).

For distance to the nearest hospital, a significant positive association was observed in Tokyo (coefficient: 6.3 × 10^−^⁶), while a significant negative association was observed in Osaka (coefficient: −6.3 × 10^−^⁶).

For distance to the nearest school, significant negative associations were found in both Tokyo (coefficient: −1.6 × 10^−^⁵) and Osaka (coefficient: −1.7 × 10^−^⁵).

For distance to the nearest station, significant positive associations were observed in both Tokyo (coefficient: 1.7 × 10^−^⁵) and Osaka (coefficient: 1.2 × 10^−^⁵).

Finally, significant negative associations between station ridership and the POC were observed in both Tokyo (coefficient: −4.0 × 10^−^⁸) and Osaka (coefficient: −3.4 × 10^−^⁸).

Model fit statistics for the fully adjusted Model 3 were: Tokyo R² = 0.28, adjusted R² = 0.28, RMSE = 0.030; Osaka R² = 0.18, adjusted R² = 0.18, RMSE = 0.043. Variance inflation factors for all models were below 1.6, indicating that multicollinearity was not a concern.

Pearson correlation coefficients between VCF and POC were: Tokyo r = 0.31 (p < 0.001); Osaka r = 0.15 (p < 0.001).

Formal interaction tests showed significant VCF × Distance zone interaction effects in both cities (Tokyo: p < 0.001; Osaka: p = 0.003), supporting the stratified analysis approach. VCF × Age group interactions were also significant (Tokyo: p = 0.008; Osaka: p = 0.015).

Moran’s I statistics on the residuals of Model 3 indicated the presence of modest spatial autocorrelation (Tokyo: Moran’s I = 0.12, p < 0.001; Osaka: Moran’s I = 0.09, p < 0.001). The implications of this finding are discussed in the Limitations section.

### Relationship between the POC and VCF based on stratified analyses by distance from the city center and school-age group

We conducted stratified analyses based on distance from the city center and school-age group, adjusting for all covariates in the same manner as in Model 3 (Table 4). As a result, significant positive associations between VCF and the POC were observed within 15 km of the city center in Tokyo and within 5–15 km in Osaka. In both Tokyo and Osaka, the estimated coefficients peaked within the 5–10-km range from the city center (Tokyo: 0.12; Osaka: 0.062), while no significant associations were observed beyond 15 km.

**Table 4:** Stratified analysis of adjusted correlations between VCF and the POC by distance zones from the city center and school-age group.

	All children		School-age groups					
			Preschool-aged children		Elementary school students		Junior high school students	
	*Coefficient* (95% CI)	*p*	*Coefficient* (95% CI)	*p*	*Coefficient* (95% CI)	*p*	*Coefficient* (95% CI)	*p*
**Tokyo**								
All zones	0.050 (0.039–0.061)	<0.0001	0.019 (0.013–0.025)	<0.0001	0.023 (0.018–0.029)	<0.0001	0.0090 (0.006–0.012)	<0.0001
0–5 km	0.096 (0.055–0.14)	<0.0001	0.059 (0.035–0.083)	<0.0001	0.040 (0.022–0057)	<0.0001	0.0076 (−0.0006–0.016)	0.070
5–10 km	0.12 (0.097–0.15)	<0.0001	0.042 (0.029–0.055)	<0.0001	0.058 (0.047–0.068)	<0.0001	0.029 (0.023–0.034)	<0.0001
10–15 km	0.049 (0.028–0.070)	<0.0001	0.023 (0.012–0.034)	<0.0001	0.022 (0.013–0.032)	<0.0001	0.0077 (0.003–0.012)	0.0013
15–20 km	0.0080 (−0.026–0.042)	0.64	−0.0017 (−0.021–0.018)	0.86	0.0082 (−0.008–0.024)	0.31	0.0042 (−0.004–0.013)	0.34
20–25 km	−0.016 (−0.058–0.026)	0.46	−0.0005 (−0.023–0.022)	0.96	−0.0027 (−0.024–0.019)	0.81	0.0008 (−0.010–0.012)	0.88
>25 km	−0.0060 (−0.027–0.015)	0.58	−0.0043 (−0.015–0.006)	0.43	−0.0009 (−0.011–0.009)	0.86	−0.0006 (−0.006–0.005)	0.82
**Osaka**								
All zones	0.040 (0.028–0.052)	<0.0001	0.019 (0.012–0.025)	<0.0001	0.020 (0.014–0.025)	<0.0001	0.0058 (0.003–0.009)	0.0004
0–5 km	0.014 (−0.026–0.055)	0.48	0.0090 (−0.013–0.031)	0.42	−0.0007 (−0.019–0.018)	0.94	−0.0038 (−0.015–0.007)	0.51
5–10 km	0.062 (0.033–0.092)	<0.0001	0.036 (0.020–0.051)	<0.0001	0.029 (0.015–0.044)	<0.0001	0.0033 (−0.004–0.010)	0.35
10–15 km	0.049 (0.027–0.071)	<0.0001	0.029 (0.017–0.040)	<0.0001	0.026 (0.015–0.036)	<0.0001	0.0043 (−0.002–0.010)	0.16
15–20 km	0.019 (−0.008–0.046)	0.18	0.012 (−0.004–0.028)	0.14	0.0082 (0.005–0.021)	0.23	0.0066 (−0.001–0.014)	0.097
20–25 km	0.037 (0.003–0.071)	0.031	0.016 (−0.002–0.035)	0.082	0.018 (0.001–0.034)	0.034	0.010 (0.002–0.019)	0.019
>25 km	0.008 (−0.033–0.049)	0.70	−0.0076 (−0.027–0.012)	0.45	0.011 (−0.010–0.031)	0.30	0.0012 (−0.010–0.012)	0.83

Regarding school-age groups (preschool-aged children, elementary school students, and junior high school students), significant positive associations between VCF and the POC were found for all groups in both Tokyo and Osaka (coefficients for Tokyo: preschool-aged children: 0.019, elementary school students: 0.023, junior high school students: 0.0090; coefficients for Osaka: preschool-aged children: 0.019, elementary school students: 0.020, junior high school students: 0.0058). The estimated coefficients for preschool-aged and elementary school children in both Tokyo and Osaka were higher than those for junior high school students.

When combining distance from the city center and school-age groups, in Tokyo, the highest estimated coefficients were observed within 5 km for preschool-aged children and within 5–10 km for elementary and junior high school students. In Osaka, the highest estimated coefficients were observed within 5–10 km for preschool-aged and elementary school children and within 20–25 km for junior high school students.

Overall, stronger associations between VCF and the proportion of younger children were found closer to the city center.

## Discussion

The main finding of this ecological cross-sectional study is that town blocks with higher vegetation cover fraction tend to have a higher proportion of children in Japan’s two largest metropolitan areas (Table 3). The relationship between the POC and VCF varied according to distance from the city center and school-age group (Table 4). Stratified analyses based on distance from the city center showed significant positive associations between VCF and the POC within 15 km in Tokyo and within 5–15 km in Osaka, respectively. In both cities, the estimated coefficients peaked within 5–10 km (Tokyo: 0.12; Osaka: 0.062), while no significant associations were observed beyond 15 km. Stratified analyses by school-age group (preschool-aged children, elementary school students, and junior high school students) showed significant positive associations in all groups (coefficients for Tokyo: preschool-aged children: 0.019, elementary students: 0.023, junior high students: 0.0090; coefficients for Osaka: preschool-aged children: 0.019, elementary students: 0.020, junior high students: 0.0058). Regarding practical significance, the coefficient of 0.05 in Tokyo means that a 0.1-unit increase in VCF (e.g., from 0.1 to 0.2) is associated with a 0.5 percentage point increase in POC. Given the mean POC of approximately 11%, this represents a modest but potentially meaningful difference at the neighborhood level.

In both cities, the coefficients for preschool-aged and elementary school children were higher than those for junior high school students. This finding aligns with those of Namizuka et al. (2022), who reported that residential priorities shift from a child-centered perspective in early childhood to parental activities and conveniences as children enter compulsory education. [28] Households with young children may initially prioritize access to urban greenery and, over time, adjust their residential preferences toward greater commuting and living convenience as children grow. When examining both distance from the city center and school-age group, in Tokyo, the highest estimated coefficients were observed within 5 km for preschool-aged children and within 5–10 km for elementary and junior high school students. In Osaka, the highest estimated coefficients were observed within 5–10 km for preschool-aged and elementary school children and within 20–25 km for junior high school students. Closer proximity to the city center was associated with stronger correlations between VCF and the proportion of younger children. These results suggest that child-rearing households with preschool- and school-aged children are concentrated in greener areas, even within the highly urbanized cores of large cities.

Other interesting findings emerged from the covariate analysis. Significant negative associations between crime rates and the POC were found in both cities, while the proportion of residential zoning showed positive associations. These findings suggest that areas with lower crime and higher residential zoning proportions tend to have higher concentrations of children [3,29].

The POC showed a significant negative association with distance to the nearest school, a significant positive association with distance to the nearest station, and a significant negative association with station ridership. These results imply that child-rearing households may avoid highly commercialized, congested areas near major stations and instead prefer quieter educational districts with good access to schools. This trend, alongside findings for VCF, crime rates, and residential zoning, highlights that child-rearing households may prioritize the quality of residential environment over commuting or daily convenience.

It is also worth noting that the association between VCF and the POC peaked in the 5–10-km zone in both cities. In Tokyo, the 5-km zone corresponds to central districts inside the Yamanote Line, such as Chiyoda, Chuo, Minato, and Bunkyo wards, while the 5–10-km zone includes areas such as Suginami, Setagaya, and Taito wards, encompassing subcenters around the city’s edge. In Osaka, the 5-km zone includes central districts inside the Osaka Loop Line, such as Kita, Chuo, Fukushima, Nishi, Tennoji, and Naniwa wards, while the 5–10-km zone includes areas such as Yodogawa, Higashiyodogawa, Abeno, Joto, and Ikuno wards. These 5–10-km zones lie just outside the dense urban cores and offer better living convenience, transportation access, and proximity to large urban parks and castles. The findings suggest that child-rearing households are particularly attracted to greener environments within these urban fringes, highlighting the potential of enhancing urban greenery to appeal to such households. Moreover, these results support the findings of Martins, who reported that urban centers and densely populated areas in Portugal suffer from severe green space shortages and adding new green spaces in these areas is particularly effective [30]

Bijnens et al. (2020) demonstrated that urban greenery positively affects children intelligence and behavior, while [31] Akpinar (2020) reported that greenery promotes physical activity among adolescents, with the lack of greenery being a major barrier. [32] Thus, the preference of child-rearing households for greener areas could reflect the well-documented benefits of urban greenery for children development and youth activity levels. Moreover, the benefits of urban greenery are not limited to children. Jiang and Huang found that frequent use of greenery within residential areas improves the life satisfaction of residents, [33] while Martins reported that the strategic placement of existing and new green spaces enhances the urban green network and provides more opportunities for outdoor activities among all age groups. [30] Our findings imply that household residential preferences adjust according to life stage priorities, balancing child-rearing needs, daily convenience, and commuting considerations. While these findings may inform urban planning discussions, caution is warranted when translating ecological associations into policy recommendations. Greening initiatives should consider potential unintended consequences, including ‘green gentrification’ that could increase housing costs and potentially displace lower-income families. Greenery enhancement should be accompanied by affordability policies to ensure equitable benefits.

It should also be noted that the association between VCF and the POC was not significant beyond 15 km from the city center. This suggests that the relationship between urban greenery and child residential concentration is not simply linear; in suburban areas, the built environment and greenery saturation may moderate this relationship. Our findings reinforce those of Wang et al., who reported that beyond a certain threshold, additional green space has limited impact on mental health. Not only green space quantity but also management quality is crucial. [34] Concerns about poor tree maintenance [35] and the importance of well-maintained spaces for encouraging children’s use and satisfaction [36] have been documented. Economic conditions, urbanization, and infrastructure investment also affect access to urban greenery [37]. Dunton et al. reported that children may avoid green spaces perceived as designed only for very young children, [38] while Jansson et al. highlighted that greened spaces, especially those with shrubs and forest like elements, promote the engagement of children with their environment. [39] Thus, the findings from our study on residential preferences by life stage and distance from the city center can provide valuable guidance for urban greenery planning and policy.

### Limitations

The strengths and limitations of this study must also be acknowledged. Unlike many previous studies based on self-reported data, we used highly reliable open datasets and conducted multifaceted statistical analyses by hypothesizing covariates.

First, a cross-sectional observational design was adopted, which limits causal inference. The observed associations could reflect household preferences for greener areas, but could also be explained by confounding factors such as housing prices, historical development patterns, or school district boundaries. Reverse causality—whereby areas with more children receive more green space investment—cannot be ruled out.

Second, several important determinants of both greenery and family residential patterns could not be included due to data availability constraints at the town block level. These include household income, housing prices, housing type/tenure, and broader socioeconomic conditions. Greener neighborhoods may also have larger dwellings or higher income levels, which could partially explain the higher POC. The reported associations are therefore conditional on the available covariates only.

Third, Moran’s I tests indicated the presence of modest spatial autocorrelation in the regression residuals. This may lead to underestimated standard errors and overly precise p-values. While spatial regression models could address this issue, the consistent significance of our findings across multiple stratified analyses and the modest magnitude of the spatial autocorrelation provide some robustness to our conclusions. Confidence intervals should nevertheless be interpreted with caution.

Fourth, the use of town blocks as the unit of analysis raises modifiable areal unit problem (MAUP) concerns. Results may differ with alternative spatial unit definitions, and within-block heterogeneity is not captured. Finer-scale analyses would be beneficial to inform practical urban greenery design policies.

Fifth, crime data definitions differed between Tokyo (all reported crimes) and Osaka (theft-related incidents only), which may affect cross-city comparisons of the crime rate covariate.

Additionally, distances were calculated from town block centroids rather than individual household locations, and only the nearest facility was considered without accounting for facility density, representing a methodological limitation. While we focused on school-age categories, incorporating additional sociodemographic factors could yield more nuanced insights. The creation of longitudinal datasets tracking VCF over time could enable causal inferences in future studies.

## Conclusions

In this ecological cross-sectional analysis of urban areas in Tokyo (4,814 town blocks) and Osaka (7,337 town blocks), we found that the residential distribution of child-rearing households is positively associated with vegetation cover fraction, even after adjusting for assumed covariates related to neighborhood environmental and transportation accessibility factors. These results suggest that the residential distribution of child-rearing households is positively associated with areas with abundant urban greenery.

Meanwhile, the relationship between the POC and VCF was found to vary by distance from the city center and by school-age group. Stratified analyses based on distance from the city center showed significant positive associations within 15 km in Tokyo and within 5–15 km in Osaka. In both cities, the highest estimated coefficients were observed within the 5–10-km zone (Tokyo: 0.12; Osaka: 0.062), with no significant associations beyond 15 km.

Stratified analyses by school-age group revealed significant positive associations between the POC and VCF across all groups (coefficients for Tokyo: preschool-aged children: 0.019, elementary school students: 0.023, junior high school students: 0.0090; coefficients for Osaka: preschool-aged children: 0.019, elementary school students: 0.020, junior high school students: 0.0058). The coefficients for preschool-aged and elementary school children in both Tokyo and Osaka were higher than those for junior high school students.

When considering both distance from the city center and school-age group, the highest estimated coefficients in Tokyo were found within 5 km for preschool-aged children and within 5–10 km for elementary and junior high school students. In Osaka, the highest estimated coefficients were observed within 5–10 km for preschool-aged and elementary school children and within 20–25 km for junior high school students. Closer proximity to the city center was associated with stronger correlations between VCF and the proportion of younger children. These findings should be interpreted within the context of Japan’s two largest megacities and may not generalize to smaller Japanese municipalities or international contexts due to unique urban forms, housing markets, and governance structures. The findings of this study on the association between urban greenery and the residential distribution of child-rearing households may provide descriptive evidence to inform the design of urban green spaces.

## Supporting information

S1 File Table S1. Descriptive statistics by distance zone from city center. Table S2. Mean population and number of children by VCF tercile. (DOCX)
