# Supplementary material for: Vegetation cover fraction and the residential distribution of child-rearing households in urban Tokyo and Osaka: An ecological cross-sectional analysis
Source: PLoS One. 2026 Mar 16;21(3):e0345197. doi: 10.1371/journal.pone.0345197 (PMC12991222; doi:10.1371/journal.pone.0345197)
Supplement: S1 File — (DOCX) [file pone.0345197.s001.docx]

**Supplementary Materials**

**Table S1. Descriptive statistics by distance zone from city center**

Note: This table shows the number of town blocks, mean VCF, and mean POC for each distance zone in Tokyo and Osaka.

**Tokyo**

| **Distance Zone** | **n (blocks)** | **Mean VCF (SD)** | **Mean POC (SD)** |
| --- | --- | --- | --- |
| 0-5 km | 608 | 0.118 (0.088) | 0.106 (0.040) |
| 5-10 km | 944 | 0.138 (0.078) | 0.103 (0.032) |
| 10-15 km | 1198 | 0.159 (0.072) | 0.113 (0.029) |
| 15-20 km | 439 | 0.212 (0.075) | 0.121 (0.030) |
| 20-25 km | 349 | 0.232 (0.084) | 0.127 (0.035) |
| >25 km | 1276 | 0.243 (0.099) | 0.121 (0.037) |

**Osaka**

| **Distance Zone** | **n (blocks)** | **Mean VCF (SD)** | **Mean POC (SD)** |
| --- | --- | --- | --- |
| 0-5 km | 731 | 0.081 (0.079) | 0.096 (0.042) |
| 5-10 km | 1481 | 0.090 (0.075) | 0.112 (0.043) |
| 10-15 km | 2158 | 0.114 (0.086) | 0.116 (0.044) |
| 15-20 km | 1363 | 0.162 (0.107) | 0.124 (0.052) |
| 20-25 km | 848 | 0.199 (0.111) | 0.125 (0.051) |
| >25 km | 757 | 0.205 (0.112) | 0.124 (0.058) |

**Table S2. Mean population and number of children by VCF tercile**

**Tokyo**

| **VCF Tercile** | **n (blocks)** | **Mean Population (SD)** | **Mean Children (SD)** | **Mean POC (SD)** |
| --- | --- | --- | --- | --- |
| Low | 1607 | 3015.2 (1766.9) | 306.8 (204.4) | 0.1003 (0.0298) |
| Medium | 1615 | 3064.9 (1769.0) | 363.3 (260.1) | 0.1173 (0.0318) |
| High | 1592 | 2336.6 (1776.3) | 295.0 (257.6) | 0.1252 (0.0382) |

**Osaka**

| **VCF Tercile** | **n (blocks)** | **Mean Population (SD)** | **Mean Children (SD)** | **Mean POC (SD)** |
| --- | --- | --- | --- | --- |
| Low | 2448 | 1101.3 (829.0) | 119.0 (103.3) | 0.1059 (0.0417) |
| Medium | 2449 | 1219.5 (1022.9) | 151.1 (160.2) | 0.1187 (0.0448) |
| High | 2441 | 1106.3 (1113.2) | 144.2 (170.1) | 0.1256 (0.0556) |

**Notes:** VCF = Vegetation Cover Fraction; POC = Proportion of Children; SD = Standard Deviation. Town blocks with VCF > 0.5 were excluded from analysis.
